# Beyond mechanistic interaction: value-based constraints on meaning in language

**DOI:** 10.3389/fpsyg.2015.01579

**Published:** 2015-10-29

**Authors:** Joanna Rączaszek-Leonardi, Iris Nomikou

**Affiliations:** ^1^Institute of Psychology, Polish Academy of SciencesWarsaw, Poland; ^2^Excellence Center in Cognitive Interactive Technology, University of BielefeldBielefeld, Germany

**Keywords:** distributed cognition, embodied cognition, values, language as social coordination, language development, dynamical systems

## Abstract

According to situated, embodied, and distributed approaches to cognition, language is a crucial means for structuring social interactions. Recent approaches that emphasize this coordinative function treat language as a system of replicable constraints on individual and interactive dynamics. In this paper, we argue that the integration of the replicable-constraints approach to language with the ecological view on values allows for a deeper insight into processes of meaning creation in interaction. Such a synthesis of these frameworks draws attention to important sources of structuring interactions beyond the sheer efficiency of a collective system in its current task situation. Most importantly, the workings of linguistic constraints will be shown as embedded in more general fields of values, which are realized on multiple timescales. Because the ontogenetic timescale offers a convenient window into the emergence of linguistic constraints, we present illustrations of concrete mechanisms through which values may become embodied in language use in development.

## Introduction

Recent research on language has made it increasingly clear that treating it as an individual computational skill (regardless of whether it is guided by innate or learned rules) is not conductive to explaining its crucial inter-individual coordinative functions. Language both distributes cognition, assigning specific roles to interaction participants, and coordinates individual resources, pooling them in task- and situation-specific synergies (Rączaszek-Leonardi and Cowley, 2012; Fusaroli et al., 2014). An explanatory framework that encompasses this coordinative property must show how linguistic structures become functionally related to the ongoing individual and interactive dynamics of action and co-action. One of the approaches that has been proposed to account for this coordinative function, which is rooted in early theories of information in biological systems, rests on treating linguistic structures not as carriers of meaning but rather as constraints—which, due to their selected controlling role, are able to influence existing dynamics (Pattee and Rączaszek-Leonardi, 2012).

Earlier work within this framework has underscored the functional pressures that shape coordinative systems through linguistic constraints to be efficient in specific tasks (Rączaszek-Leonardi and Kelso, 2008; Fusaroli et al., 2012; Rączaszek-Leonardi, 2014; Zubek et al., under review). The goal of this paper is to show that the sources of constraints cannot be limited to the sole efficiency of a distributed system in dealing with immediate situations. Using the ecological approach to cognition (Gibson, 1966, 1979) and value-realizing theory (Hodges and Baron, 1992; Hodges and Geyer, 2006; Hodges, 2014; Hodges et al., 2014), we will show that language is not only a form of functional, task-oriented coupling but also, or perhaps above all, a value-realizing activity using value-preserving structures.

To realize this goal, we will briefly present the approach to language as a system of replicable constraints, which has been developed on the grounds of information theory in biological systems proposed by Howard Pattee, among others. Next, to deepen and broaden this approach, we will turn to the work of Gibson on affordances, developed further by Hodges and Baron (1992), in which the authors show the fundamental role of values in psychological theory. We will briefly present their proposal of how to avoid strictly mechanistic (law-like) or strictly algorithmic (rule-like) accounts of human behavior by considering a more general value-realization frame.

The two theories together will provide a background for demonstrating that linguistic constraints emerge in such a way as to preserve both general systemic values (such as efficiency and coherence) and culturally selected values (such as, e.g., specific social structure). Uncovering the processes through which linguistic expressions are endowed with value-preserving and controlling power over interaction requires attention to processes on several distinct timescales (e.g., on-line, developmental, and cultural and biological evolution). Here we will focus on the developmental timescale to show, through a microanalysis of instances of language use in mother-infant interaction, the ways in which such value-preserving constraining powers might arise. Conclusions concern the usefulness of such a framework in systematizing the complex interplay of forces in linguistic coordination and guiding the search for both important values that may be present in interaction and mechanisms through which they are preserved.

## Language as a system of constraints controlling social interaction

In recent years, language has increasingly been treated as primarily a sophisticated mean of social coordination. On such view, the “mapping” or referential function of language for an individual mind with respect to the world becomes less important than, or subservient to, its role in controlling collective systems formed through human interaction (Rączaszek-Leonardi and Cowley, 2012). This change in views on language is a part of the broader shift in the theory of cognition: from considering it as a form of solitary information-processing by an individual mind/brain to regarding it primarily as a basis for embodied, situated, distributed and adaptive action.

This interactive and collective dimension of language functioning, pertaining to the coordination of global systems (such as dyads, groups, and societies) over longer timescales, must be integrated with more local and individual skills that lead to language production and understanding. Recent years have seen several attempts to clarify the relations among the multiple systems and multiple timescales relevant to language, both in empirical studies of language and in computational simulations of emergent linguistic structuring and human performance (Smith et al., 2003; Van Orden et al., 2003; Rączaszek-Leonardi, 2010; Wallot and Van Orden, 2011a,b; Dale and Lupyan, 2012). Such frameworks seem to provide a broader repertoire of useful explanatory concepts that can be applied to emergent language structures, the manner in which they are learned, and their functioning in interaction. Traditional cognitive models locate the forces responsible for linguistic behaviors or linguistic structures within the mental machinery of an individual, but in the embodied, situated and distributed approaches, the sources of the forces are many. These forces arise from the requirements of stability, functionality and learnability of the system, shaped in multiple interactions over various periods of time.

Thus, one problem for theories of language within such a coordinative framework is its complexity, its dependence on processes on multiple timescales and within various systems. However, an even more pressing issue concerns the relation between informational (“symbolic”) structures and the multiple dynamical events that contribute to individual cognition and action as well as inter-individual interactive processes. In other words, in the framework where the controlling role of language becomes at least equally important as the referential one, the relation between the symbolic and dynamic aspects of language has to go beyond the traditional “mapping” relationship.

One of the approaches that has been developed specifically to address this problem has grown out of theories of information in biological systems. Following work in philosophy of biology (Polanyi, 1968; Pattee, 1969, 1982), this framework treats informational structures as replicable constraints that render various kinds of dynamics functional in a given environment by reducing selected degrees of freedom of an active system. Within this approach, physical informational structures are inseparable from dynamical events on many timescales: they arise from them and they are selected to have a controlling role with respect to them (e.g., Pattee, 1969, 1982).

This approach, as applied to natural language, has been presented in detail elsewhere (Pattee and Rączaszek-Leonardi, 2012; Rączaszek-Leonardi, 2014). To briefly restate its main tenets, let us underscore the change it brings to the nature of linguistic structures: they become informational not because they map onto content in the mind or objects and events in the world but because they have a constraining role, which renders the coordination of individuals appropriate and flexible under environmental demands. This constraining role is established through selection during a particular history of the inter-individual coordination in a given environment and within a given culture. Such a perspective underscores the fact that language consists of physical events, having a causal role with respect to the dynamics of action or co-action. However, the crucial aspect of this role is not only that it is (physically) causal but also that it is selective (Rączaszek-Leonardi, in press): due to its history within a system, an informational structure evolves as a specific constraint, which is able to bind selected degrees of freedom to render the behavior of the system adaptive.

The study of linguistic structures is thus best carried within dynamics of interaction, i.e., in their “natural habitat” (Schegloff, 1996). The interactive dimension of linguistic processes comes to the fore. The systemic-level qualities can be the substrate for selection of useful structures in their own right (Smaldino, 2014). Utterances are informational because they have been selected in cultural evolution to influence interactions in a particular way and because individuals learn to perceive them and use them as effective controls.

There is a strong compatibility between such a view on the informative properties of linguistic structures and the approach to perception and action within ecological psychology. According to this approach, aspects of the environment are informational because organisms are tuned to them in evolution, development and experience in a way that selectively constrains their actions (i.e., these aspects of the environment become affordances). In a similar way, language can be considered a system of interactional affordances, directly perceived as opportunities for action, enabling interaction by mutually constraining the participants (Worgan and Moore, 2010; Rączaszek-Leonardi et al., 2013; Rączaszek-Leonardi, in press).

So far, however, according to views of language as social coordination, including the replicable-constraints framework, the efficiency of coordination seemed to be the most important, if not the only, decisive factor for the selection of controlling structures. The application of such frameworks in empirical research most often concerned task-oriented dialogues, with quantitative measures of performance on the task serving as indices of “good” or “successful” communication (e.g., Fusaroli et al., 2012). A major concern arises that such an emphasis might lead to a mechanistic and simplistic view of the coordinative role of language.

Many traditional, functionally oriented approaches to language underscored that the immediate functional or informative role of language is but one aspect of its social and coordinative role (Linell, 2009; Goodwin, 2013). The fields of language socialization, conversation analysis and ethnomethodology have traditionally focused on uncovering not only *what* is achieved within interaction but also *how* it is achieved. Contributions in the field have revealed how social organization is constructed (and preserved) not only through the use of linguistic resources but also through patterns of body alignment, gaze, and prosody (e.g., Goodwin and Cekaite, 2013). An extensive amount of work has been devoted to understanding how social relationships are constituted within everyday interaction and language use (e.g., Goodwin, 1990; Stivers et al., 2011; Enfield, 2013; Lindström and Sorjonen, 2013) and how values, ideologies and socialization goals inform ways of interacting (e.g., De León, 2000; for a collection see Duranti et al., 2012). These analyses of multiple social “agendas” in communication depict language as a constitutive element of social practice, which preserves social structures and a mode of preservation of social order.

By taking social action as the main analytic objective, these approaches are especially powerful when considering language in the developmental timescale (Wootton, 2005; Kidwell, 2013; see also Gardner and Forrester, 2010). The functioning of language in development is perhaps a particularly vivid example of how rich the role of language is in social coordination, making evident the multiple aspects of coordination that language might influence. Through linguistic interactions, a child learns not only how to name things (which seems to have been the main emphasis of research in the past several decades, e.g., Golinkoff et al., 1994; Smith et al., 2002) and how to direct attention to others and objects but also—crucially—how to enter interactions. This includes learning how to adapt to a communication partner through temporal matching of activity level, facial expressions, and properties of vocalizations (Stern, 1974, 1985; Trevarthen, 1979; Beebe et al., 1982; Papoušek and Papoušek, 1989; Leimbrink, 2010) and how to keep a common rhythm of speech and action (Jaffe et al., 2001). It also includes learning to be a part of a distributed system, i.e., learning about possible roles that the child can take on in common endeavors (Gratier et al., 2015; Nomikou et al., under review); learning to expect and project unfolding sequences of actions in common endeavors (Heller and Rohlfing, 2015); and stabilizing conventionalized social routines (Strähle, 2013).

The role of very early linguistic interactions is thus not the practical efficiency of a given co-action (e.g., getting a child dressed or fed), nor it is the assumed “endpoint” of language learning which, by some accounts, consists in mapping linguistic structures to the external world. Language, from an early age, takes on a role of subtle interaction control, shaping interactions into socially acceptable coordinative systems, and as studies analyzing talk-in-interaction show (Ochs et al., 1996), it remains so in the adult world. The question arises: how to introduce this richness into the framework of language as a system of replicable constraints to save it from the view of mechanistic functionality? How can one introduce criteria that make interactive human systems stable and coherent in the long run, as parts of broader environment-organism systems? In other words, an integrated theory should account for the fact that social coordinative systems are not only devices, realizing “functions” but also self-sustaining systems in a particular niche, which is a crucial source of the values to be realized.

## A framework for multiple-values realization

The long-term integrity of a system in the face of environmental challenges, apart from current functionality, is thus an obvious candidate for another value realized on many timescales. It can be in conflict with the value of immediate efficiency, because, for example, it may promote sameness in participants when divergence would be more functional. How linguistic constraints function in this broader field of values, and what the role of values is in shaping these constraints, can be better understood by reaching deeper into the theoretical resources of ecological psychology, which, among very few approaches in scientific psychology, has sought to give values an important place in its explanatory framework. If linguistic constraints indeed arise in a similar way as affordances, by developing and tuning sensitivities to directly pick up what they specify (Rączaszek-Leonardi et al., 2013), then, similarly to affordances, they are saturated with values, and their direct apprehension enables value realization.

Ecological psychology has always shown sensitivity to the social and moral dimensions of organisms acting in the world, both in its beginnings (e.g., Gibson and Crooks, 1938) and especially in its later developments toward social ecological psychology (e.g., Hodges and Baron, 1992). Criticizing traditional approaches to psychology (both the behaviorist and the information-processing paradigms), Ed Reed wrote:

“*To the extent that our psychologies ever deal with important issues of value or meaning, they are nonnaturalistic, or even downright non-scientific. Conversely, to the extent that they have been scientific, our psychologies have had little or nothing to say about meaningful aspects of life.”*

Reed (1996 p. 96).

One of the goals of the ecological approach to psychology was to preserve both scientific rigor and the possibility of referring to meaning and values to move toward socially responsible science (Hodges and Baron, 1992). Gibson, moving away from a “psychology of the stimulus,” sought to frame organisms as active, value-realizing parts of the world instead of stimulus-driven information processors.

In a seminal paper, James J. Gibson and L. E. Crooks analyzed an everyday action, driving a car, from the perspective of the field of forces that motivate its ultimate outcome. We usually think about such activities as determined by the physical properties of objects, artifacts and surroundings, as well as by the set of conventional rules of traffic regulations, meanwhile overlooking other powerful and pervasive constraints. A car will be stopped by a person standing on the road: this will not be due to an arbitrary rule (as in the case of stopping at a red light), nor will it be the result of physical laws that impede motion (as in the case of encountering a brick wall) (Hodges and Baron, 1992).

What Gibson and Crooks have shown is that during the act of driving, multiple values are simultaneously realized, which stem neither from the physics of the situation nor from codified rules, but which influence the direct pickup of information from the environment. For example, the seemingly straightforward time-to-contact estimation seems to be strongly influenced by individual differences in valuing safety. Other values—such as speed, accuracy, trust in others and in infrastructure, and respect for other users of the road—are also present in driving. Note that almost all of these values are social; that is, they depend on relations to others, which makes driving a rather bad example of a solitary activity, something it is often thought to be (Gibson and Crooks, 1938). Gibson therefore was after “meaning and value of a new sort.” Objects are defined in terms of “ecological physics,” not “physical physics,” as they “possess meaning and value to begin with” (Gibson, 1979). Due to way perceptual systems evolve and are tuned through experience, “‘values’ and ‘meanings’ of things in the environment can be directly perceived” (Gibson, 1979, p. 127), not requiring complex mental machinery and extensive explicit knowledge.

In their 1992 paper, Hodges and Baron further developed arguments for the importance of values in ecological psychology, linking it more firmly to the agenda and traditions of social psychology. Congruently with Gibson's approach (and unlike most traditional approaches to social and moral psychology), they also do not place values in a mentally represented system of rules. Values, they claim, seem to be more law-like than rules, yet also more rule-like than laws. They are not laws because they are not as unavoidable and unintentional as laws: there is a (slight) choice as to which value to follow if multiple values are in conflict. They are not rules, either, because rather than being mental constructs, values can be picked up directly in the act of perception. They are enacted and embodied in social practices and artifacts, and they directly specify actions. Thus, values are also more obligatory than rules, which are easy to break, as they usually overdetermine behavior (e.g., “keep quiet!”), and they appear where there is a real choice of behaviors.

Both lawful (in terms of “ecological physics”) and rule-following behavior should be seen as “nested within a psychology of values.” The public and perceivable values are not just “appended” to a lawfully functioning system (Turvey, 1977, after Hodges and Baron, 1992) but rather permeate every available affordance. Perception is therefore veridical about utilities, possibilities and values, and only together do these properties become affordances.

### Omnipresent, transparent, biasing

Hodges and Baron point to the omnipresence of values and their realization in deeds, ways of behaving, social and cultural customs, habitat arrangements, and artifacts. They quote Valsiner's (1987) research on socialization at mealtime and interpret it in terms of value-realization. Not only the customs at table—the culturally appropriate use of dishes and cutlery that the child learns—but also the designs of tables and artifacts embody a multiplicity of values. The values range from safety and economy in using resources to maintaining proper social relations (e.g., by a child's being seated on a highchair to make him or her appear “equal” to others) to comfort and physical convenience. These values are constantly in tension and constantly renegotiated. This also means that the child must be given freedom to explore and test boundaries.

An interesting observation is that the design of objects used at the table makes their “proper” means of use easier. Thus, values are also present in the shapes of objects (for example, the shape- and weight-balance of cups, which afford drinking and repositioning without spills). The remaining freedom allows the child to learn that she can be an agent, which leads to the realization of other values, such as responsibility. The child's caregivers are thus neither passive observers nor active feeders; rather, they allow the child to make choices to navigate convenient and acceptable ways of acting.

Let us underline again that most of those values are never made explicit. They are non-conscious and transparent (in the sense that it is difficult to notice them), pervading the structure of objects, actions and events, and being enacted unreflectively together with the child rather than being thought about and taught overtly. This, as ecological psychologists often note, makes it tricky to control for them in experiments, as they are often transparent to researchers, too. The experiment is a highly structured social situation, and obliviousness to this fact often skews the interpretations of results. As Van Orden and Holden wrote: “*Any credible research program should begin with a plausible story of how laboratory protocols yield cooperative performances*” (Van Orden and Holden, 2002, p. 102). For example, if a participant in an fMRI experiment is asked to tell the truth 50% of the time and to lie the other 50%, then he is truthful to the experimenter's demands when fulfilling this request. Are we then measuring the neural correlates to an act of “real” lying, as declared in the goal of the study? Would the participant's brain become similarly activated if he were really deceiving the person with whom he collaborates within the experimental situation?

A great example of discovering the landscape of values that people bring with them to the laboratory situation, and of how ignoring them leads to misinterpretation of the results, is Hodges and Geyer's reanalysis of the famous Asch “conformity” experiment (Hodges and Geyer, 2006). The study (Asch, 1956) required participants to judge the length of a line in the face of previous, often untruthful, judgments from the group that the participant was part of, which consisted of the experimenter's confederates. The results, which show that people sometimes misjudge length in accordance with majority opinion, have usually been interpreted as showing the undesirable conformity of human beings and their inability to withstand social pressure. What Hodges and Geyer have shown is that the distribution of assents and dissents is better seen not as simply compromising the truth for the sake of conformity, but rather as testifying to the attempts of preservation of several values beyond that of “truthfulness.” In this particular experimental setting, in which a group was repetitively engaged in a common task for an extended period of time, the coherence of the group was another very important value. Participants might have maintained this coherence by assenting to the group's judgment to communicate the acknowledgment of its members' opinions. After all, each time one communicated the truth to the experimenter (the real length of the line) in the face of the others' differing opinion, one also communicated distrust in the others and a lack of respect for their judgments. As Hodges writes, the participants in the Asch's experiments, complying sometimes with the wrong judgment of the group, “gave evidence of caring for truth, caring for others and caring that others care for the truth.” A careful analysis of the participants' responses revealed that indeed the majority of the participants agreed with the erroneous judgments only 25–30% of the time. What is even more interesting, they distributed their conforming responses over the trials in such a way as to maintain a good relationship with the group over time.

Because values are transparent, multiple, and present in every detectable affordance, linguistic constraints, which are “manufactured” over the timescale of cultural evolution to effectuate good coordination, must also incorporate values. What will be shown next is that by naturally embedding linguistic structures in embodied interactions over many timescales, the replicable-constraints approach to language might be a useful one for research on values. It may offer new perspectives on how this incorporation might happen—that is, on the evolutionary, cultural and developmental mechanisms that lead to sensible and value-preserving reductions of degrees of freedom of the systems that arise from coordination. On the other hand, the value-realizing theory shows how linguistic constraints are nested within a more general field of values and through this helps identify language's broader ecology.

Seen from this perspective, the timescale of language acquisition might be particularly interesting for the study of how dynamics on the individual and interactional levels become constrained by culturally stabilized ways of co-action and language use. This perspective allows us to look at this process as something more than the “acquisition of rules” by a child, and it will more likely reveal the multiplicity and complexity of motives for the particular form of interaction to emerge or the particular structure to stabilize. As Hodges and Baron emphasize: “*Social learning is inevitably moral, in an elementary sense of the term, and it is probably a mistake first to construct a behavior theory without reference to social interaction, and then to attach it only at the end*” (Gibson, 1950, p. 155, after Hodges and Baron, 1992).

## Linguistic constraints are nested within fields of values

The replicable-constraints approach to language advances a complex, history-dependent and systemic ontology of constraints. These constraints are structures that accrue controlling powers over interactions within cultural systems over the timescales of biological and cultural evolution and development. Values provide more general constraints or fields in which social systems are embedded (Hodges and Baron, 1992). Thus, they can be likened to globally acting constraints, or to boundary conditions of dynamical systems (here: social systems) within which other constraints emerge (Hodges, 2007).

There are at least three tenets of the constraints approach to language that make it particularly compatible with the ecological approach to values. The first one is the systemic and dynamic view, which sheds light on the place of values and the ways in which they can be identified. Values influence where and which constraints will arise to limit the dynamics of a system; they change the relative probability of one constraint's being selected over another. As stated above, this systemic view underscores a concrete important value that is distinct from efficiency of coordination in a particular environment: upholding a coherent system, structured in the process of adaptation to environmental demands becomes equally essential.

The second tenet is underscoring the importance of timescales. Doing so may help to show how realization of the same values may be achieved by different mechanisms, depending on the systems and timescales to which they pertain. For example, the value of the stability and coherence of interactive systems is achieved not only through slowly evolving mechanisms that influence the shape of our body and ensure automatic attention to particular properties (such as eyes, gaze, biological movement, etc.) but also through more rapid education of action and perception in ontogeny, which helps to maintain systemic coherence within particular cultural schemes of co-action. Finally, coherence is maintained in the timescale of current behavior by, e.g., moving and speaking in a particular way, or by conforming sometimes to the opinions of others, as in the example discussed above.

The third tenet brings attention to the importance of the physicality, externalization, and replicability of constraints. As briefly explained above (and in more detail in, e.g., Pattee and Rączaszek-Leonardi, 2012), the repetitive physical presence of an utterance (or a written mark) is necessary to maintain and reenact the causal role of a constraint. The stabilized form of this reenactment, which depends on the system's history, integrates the constraining power of language over slower timescales. Similarly in the case of values: selective reenactment, along with the specific “morphology” of behavior, is the main measurable proof of their presence.

A vivid example of such presence of values in movement (and of their readability form movement) is Hodges and Lindhiem's study on carrying babies as opposed to groceries. The authors show that people move differently, perceive steps as less steppable and are more cautious when hopping over gaps when carrying a baby than when carrying a potato bag. Our body's movement reveals to observers the value of what we are carrying (Hodges and Lindhiem, 2006). The “moral order” is “dramatized and made salient in everyday practices” (Macnamara, 1991, after Hodges and Baron, 1992). Again, it is not simple rule following and rule enforcement that do the trick, as values are not easily codified and frequently enacted unconsciously. Often it is our body, and often only after the fact, that tells us that some values were strongly conflict or that they were violated. As Cuffari notes, to discover this, we should investigate “underexplored but highly relevant dimensions of our embodiment, including bodily protest, dissonance, discomfort, difference, and betrayal” (Cuffari, 2014, p. 3).

The necessity of a physical manifestation of a value—through an utterance, a way of uttering, a trajectory of movement, or through timing in interaction (i.e., in general, through modes of re-enaction)—draws attention to different possibilities of identifying and studying values. Reification of a value in the form of a mental rule does not seem necessary, or indeed might be detrimental for its investigation. A formalized, codified, conscious value is no longer a value: it becomes a rule—less obligatory and easier to break. Thus, rather than guiding researchers to the study of mental contents, these assumptions guide them to the microanalyses of interactions, timing and trajectories of actions and co-actions as well as the forms and modes of use of artifacts. The theoretical informativeness of such external manifestations offers a strong hope for the possibility of measurable variables and a rigorous study of values.

The constraints approach with the above three tenets (systemicity, timescales and physicality of enactions) may order, systematize and offer new perspectives on how value-skilled perception emerges—that is, on how, on different timescales, values seep into and shape every perception, move, artifact and niche we construct. The question of the origin of values is obviously too difficult to tackle here. However, the framework offers a glimpse of the variety of possible pressures and their respective timescales: Some of the value constraints arise on the timescale of biological evolution, stabilized in the shape of our body, with its intentional form (Merleau-Ponty, 1962) being “about” its proper relation to the world and to conspecifics. Others emerge as very basic, inborn mechanisms involved in orienting to the others, constituting ingredients of “social glue.” However, still others arise in development as our perception and behaviors are shaped within interactions in which cultural values are enacted.

## Meaning: It happens all the time—an illustration in development

The developmental timescale is particularly relevant for demonstrating how without explicit education, many values are learned in action and joint action. As already discussed above, values are materialized not only in the use of linguistic resources but also in body alignment, timing, gaze, and prosody.

Social scripts are enacted, and proper participation in them is enforced and reinforced (Rączaszek-Leonardi et al., 2013). The same is true for language, which is apprehended in interaction. The affordances of utterances arise in enactments in a value-preserving way. How language structures are selected and what they mean thus depends on the invisible network of values that limit possible choices. Values decide whether particular uses of language will become interactional affordances and what exactly they would afford. Grammatical structures may take shape under the pressure of social structure, which, e.g., influences patterns of turn-taking, and in turn, those grammatical structures will uphold and stabilize these patterns (Ochs et al., 1996; Tanaka, 1999).

In the view of language as a system of replicable constraints, as briefly explained above, linguistic structures accrue their controlling power over multiple interactions. This requires their repetitive involvement as parts of structured episodes. After a history of enactions within a caregiver-infant dyad, an utterance gains the power to bind certain degrees of freedom of a dyadic system, making certain behaviors more probable than others. What is important is that unlike in the traditional views of language and language acquisition, an utterance is often neither “pointing to” nor referring to any specific object or action. The special timing of its production within an interactive episode, anchored to specific movements in interaction, makes it effectuate control over it, binding the participants' behaviors to a situation and being able to evoke the coordinative pattern when needed.

In the examples that follow, we illustrate one of the ways in which values may enter into and modify the perceptual and behavioral repertoire. The proper ways of coaction involving language use is shaped under pressure form multiple values, the efficiency of a coordinative system in the physical world being only one of them. Another strong source of values is systemic coherence, by which we mean not only a simple affiliative coherence but also a socially appropriate one, which preserves the social structure of the collective over the long term, and which may influence the perceived structure of a task. Our examples will also illustrate how values structure interactions in a more “arbitrary” way than laws, leaving more of a choice but, on the other hand, doing so in a less idiosyncratic, less explicit and less codified way than rules.

Both examples come from the video corpus of mother-infant interactions collected in a specific situation involving diaper changing. For a detailed description of the corpus see Nomikou and Rohlfing (2011). With the present case analyses we illustrate the way in which values are being preserved in co-action and language use and highlight *how* they are preserved by the particular timing and structure of movements, vocalizations and utterances.

### Example 1

**Figure 1:**
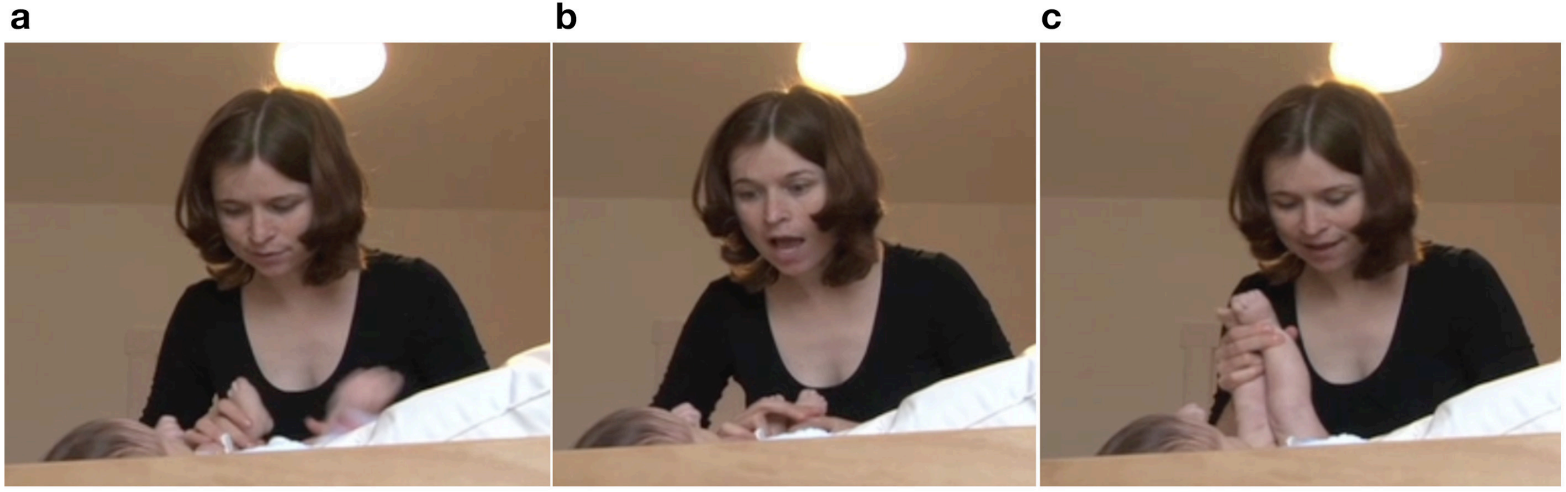
**Video grabs corresponding to Example 1, transcript lines 1 and 2**.

In this fragment, the mother initiates an utterance in line 1, while looking down at the infant's belly as she engages in the diapering activity (Figure 1a). In line 2, the infant vocalizes before the mother has concluded her utterance. The mother immediately interrupts her utterance. She quickly switches her gaze toward the infant to attend to the infant's vocalization. She does so by raising her eyebrows (Figure 1b) and looking at the infant with an activated face throughout the infant's two vocalizations. She then looks back down, returning to the diapering action (Figure 1c). Her reaction here indicates that she values the infant's vocalization as important. Not only does she stop to “give the floor” to the infant, but her facial expression and gaze also reveal that she is giving her whole attention to the infant's initiative.


1 M: na [du^∧^]^(a)^
    *so you*
2 I:[<vocalization>^(b)^
     (0.6s) <vocalization>^(c)^(0.5 s)=
      =[<vocalization>


**Figure 2:**
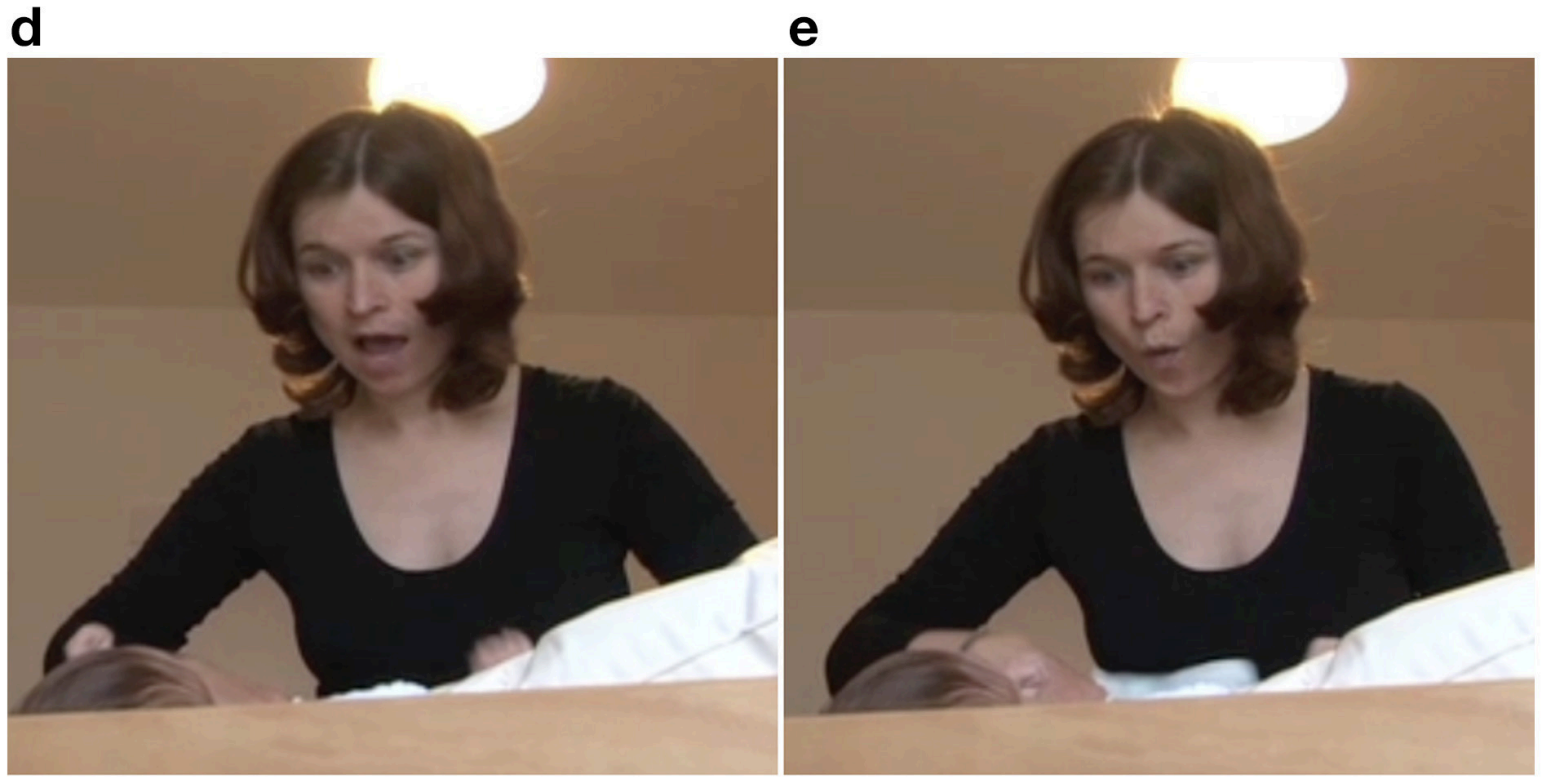
**Video grabs corresponding to Example 1, transcript line 3**.

In line 3, the mother comments on the infant's vocalizations, thus acknowledging them as something worth talking about. While the mother is speaking, the infant vocalizes in an overlapping manner (line 4). The mother pauses (line 3) and acknowledges the vocalization of the infant by engaging once again in a sudden change in her facial expression (Figures 2d,e). She again looks at the infant's face and raises her eyebrows and opens her mouth, thus being attentive to the infant's vocalization (Figure 2d) and reacting with an expression of astonishment. She then produces a prolonged “oh” while continuing to engage in an exaggerated facial expression (Figure 2e).


3 M: [erzählst du was?^(d)^ (1.2 s) [o::]:h^(e)^
                                (0.8 s) was=
    =erz[ählst denn du] der Mutti (.) hm?
    *are you telling something?(1.2 s)*
                            *[o::]:h (0.8 s)=*
    *=what are you telling mommy (.) huh?*
4 I: [<vocalization>]


**Figure 3:**
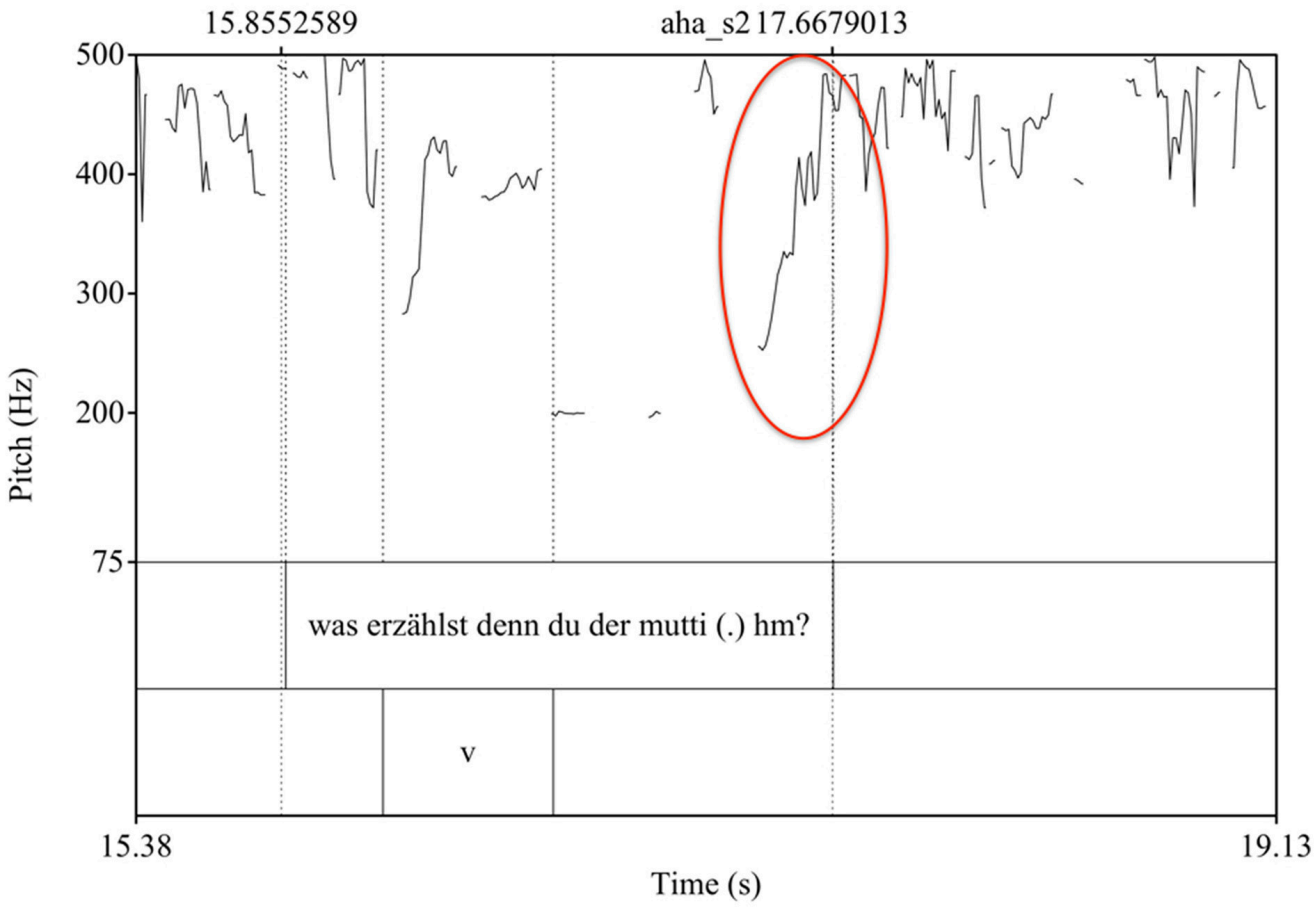
**Pitch curves from Praat**. The rising intonation of “*huh”* in Example 1 line 3 is marked in red.

**Figure 4:**
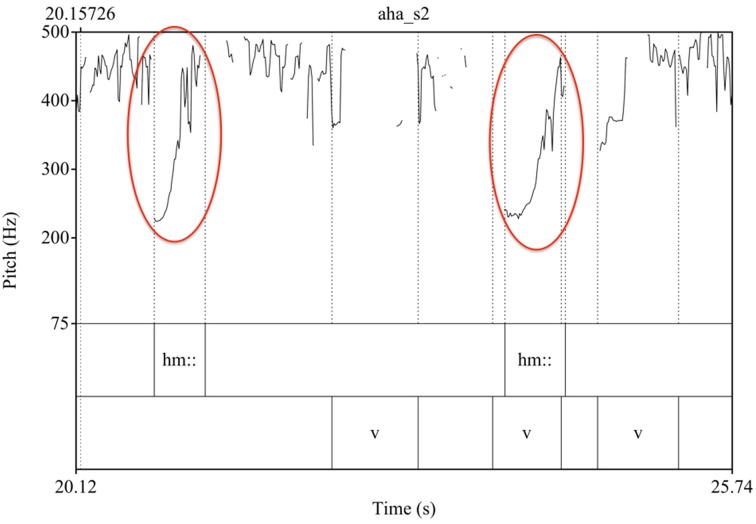
**Pitch curves from Praat for Example 1**. The rising intonation of the tag question “*huh,”* which is repeated twice in the mother's attempt to elicit and respond to the infant's vocalization, is marked in red.

Also of interest here is the use of prosody. In line 3, the mother ends her utterance with a tag question, “*huh,”* in which she uses an intonational contrast—namely a rising pitch (see Figure 3)—making a response from the infant relevant. After a long pause in line 5, the mother continues her attempt at eliciting a response by repeating the same intonation pattern (line 6), while in line (10), she responds to the infant's vocalization with the same intonation pattern (Figure 4).


5 (3.2 s)
6 M: hm::?
7 (1.1 s)
8 I: <vocalization>
9 I: [<vocalization>]
10 M:[hm:::?]


Next, the mother responds to the infant's vocalization by looming over the infant and producing a vocalization, which imitates in quality the vocalizations of the infant. In this part of the sequence, the infant's vocalizations are treated as contributions to the interaction. By mirroring the infant's behavior, the mother is responding in a way that makes the infant's actions “sensible” for the interaction. Here, the turns are constructed by using the materials the infant provides and then building upon them. Thus, the infant's vocalizations have an effect on the interaction.

**Figure 5:**
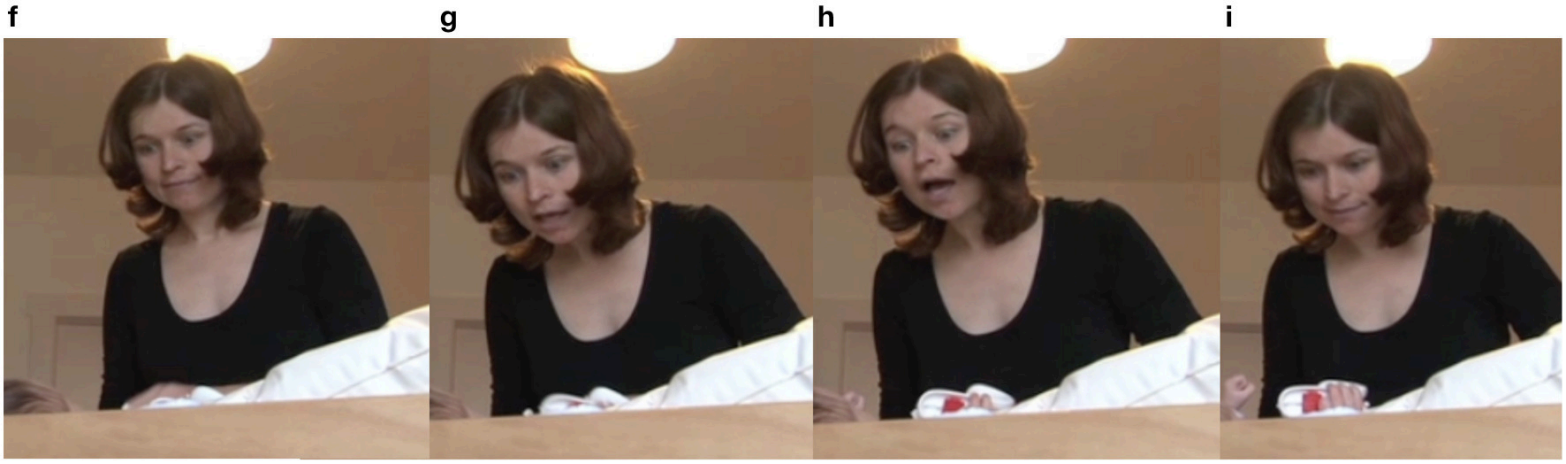
**Video grabs corresponding to Example 1, transcript lines 25 and 26**.

Later in the sequence, in line 25, after a relatively long pause (2.1 s), the infant produces two vocalizations separated by a short pause. The first of the vocalizations is longer than her other vocalizations (2.1 s), and it also has a vocalic quality, in contrast with her other vocalizations in the sequence, which are mostly consonantal. Figures 5f–h illustrate the mother's reaction to this vocalization. In Figure 5f, the mother is looking up to the infant. In Figure 5g, the mother is intensifying her display of attentiveness by looming in on the infant and moving her head forward. In Figure 5h, the mother initiates an upward half-nod, preparing to say the “aha” that follows in line 26, but she holds the position for the duration of the vocalization, waiting for the infant to speak out before she reacts, thus making the infant's contribution even more valuable. In line 26, she utters the prolonged continuer “aha” (eng. “uhum”) and returns to a neutral position (Figure 5i).


24 (2.1 s)
25 I: <vocalization>^(f, g)^(0.5s)=
      = ^(h)^<vocalization>
26 M: ah[a]?^(i)^


From the above fragment, we see that practically all of the infant's vocalizations are treated with attention and embedded in a turn-taking-like structure. What is even more interesting is that the mother provides such an intense reaction—both in terms of bodily resources activated and in terms of a prolonged continuer—to an utterance consisting of vocalic vocalization and thus more language-like than a consonantal vocalization. This behavior could thus be an example of how values, or the different weighing of the repertoire of available resources, might shape the development of language—in this case, the development of preverbal vocalizations, which will eventually lead to the production of words.

The example above dealt predominantly with the proper structuring of linguistic exchange and with acknowledging the infant as an active partner in shaping interaction. Agency and responsibility for actions seem to be frequent values that impinge on and are maintained by the ways of coaction, as illustrated by the next example.

### Example 2

**Figure 6:**
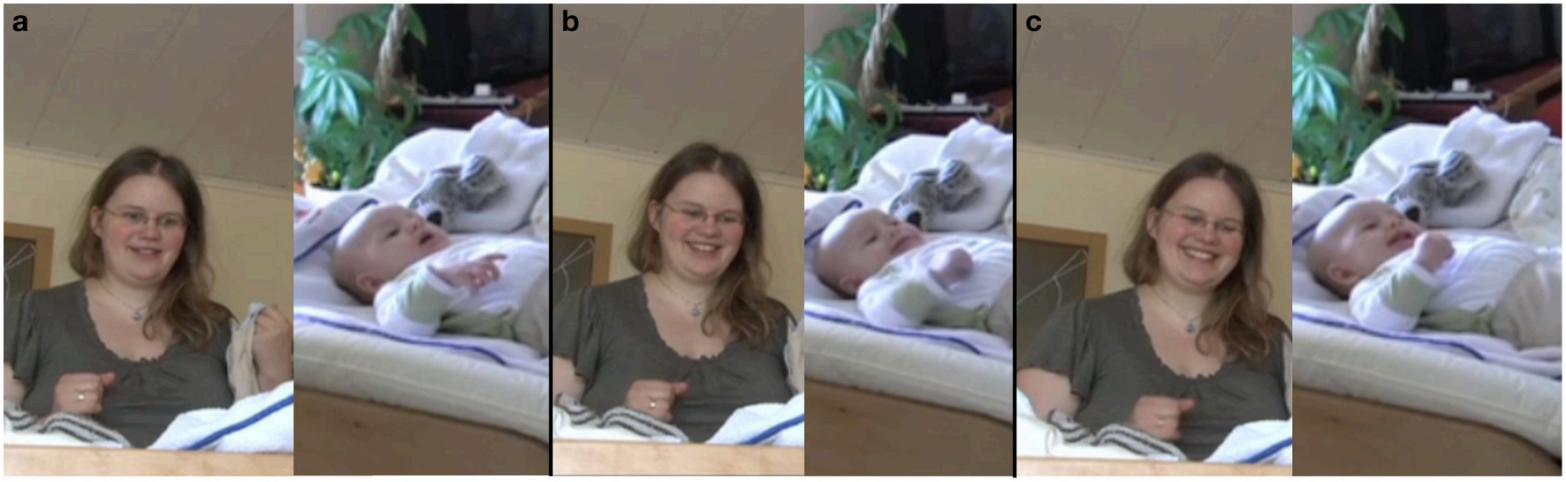
**Video grabs corresponding to Example 2, transcript lines 1 and 3**.

The sequence begins with the infant vocalizing (line 1) and wiggling his legs. In line 2, the mother pauses abruptly, stands in front of the changing table and initiates a repair with the word “what” (see Drew, 1997). At this point, mother and infant are looking at each other with neutral facial expressions (Figure 6a). After a pause (line 3), the mother repeats the repair initiator “what” (line 4). At this point, we can see that both mother and infant are starting to smile (Figure 6b). In Figure 6c, a fully developed mutual smile appears, and only then does the mother return to her activity, by announcing the next action step (line 4). This sequence makes visible the collaborative construction of the interaction.


1 I: <vocalization>
2 M: was?^(a)^
     *what?*
3 (1.5 s)
4 M: ^(b)^was?^(c)^ (.) ich zieh mal die hose an
     *what (.) I will put the pants on*


In line 1, the mother cannot interpret the infant's vocalization. She initiates a repair, displaying her doubt with a neutral facial expression, while simultaneously aligning herself with the neutral expression of the infant. In line 4, mother and infant are moving to another interactive state; namely, they are initiating a smile. It seems that it is only when they both reach a point of producing a big smile that the activity is resumed. This behavior can be interpreted as a striving for mutual acknowledgment of participation in interaction.

**Figure 7:**
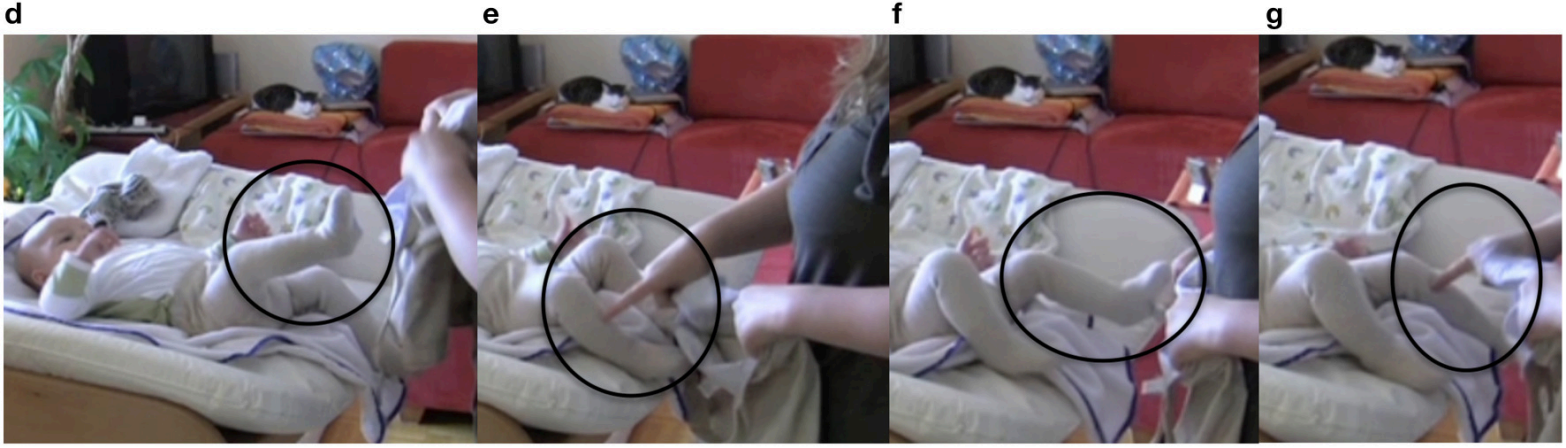
**Video grabs corresponding to Example 2, transcript lines 5–8**.

During the pause in line 5, the infant stretches one leg (Figure 7d). In line 6, the mother responds to this movement by asking the infant whether they should start with that particular leg. She points to and touches that leg in synchrony with the deictic “this” (Figure 7e). Next in the sequence, the infant stretches the other leg (line 7, Figure 7f). The mother again responds with the same pattern, pointing to and touching the other leg in synchrony with the deictic “this” (Figure 7g).


5 (2.7 s)^(d)^
6 M: mit dem ^(e)^bein?
     *with this leg?*
7 (1.5 s)^(f)^
8 M: oder mit dem ^(g)^bein?
     *or with this leg?*


In this sequence, the infant's stretched leg is treated as a proposal and an affordance for the next action sequence. This instills in the diaper-changing activity the value of it being a collaborative task in which proposals are made—in this case, the infant's leg movements, which are acknowledged as fully legitimate, “authored” input from the infant to the co-constructed interactive event. This is made perceivable by the mother's verbalizing the action of the infant presented as questions in lines 6 and 8 and by her synchronizing her speech with her pointing gesture, providing a tactile sensation to the deictic expression “this” (see also Nomikou and Rohlfing, 2011) for an analysis of action-language synchrony).

**Figure 8:**
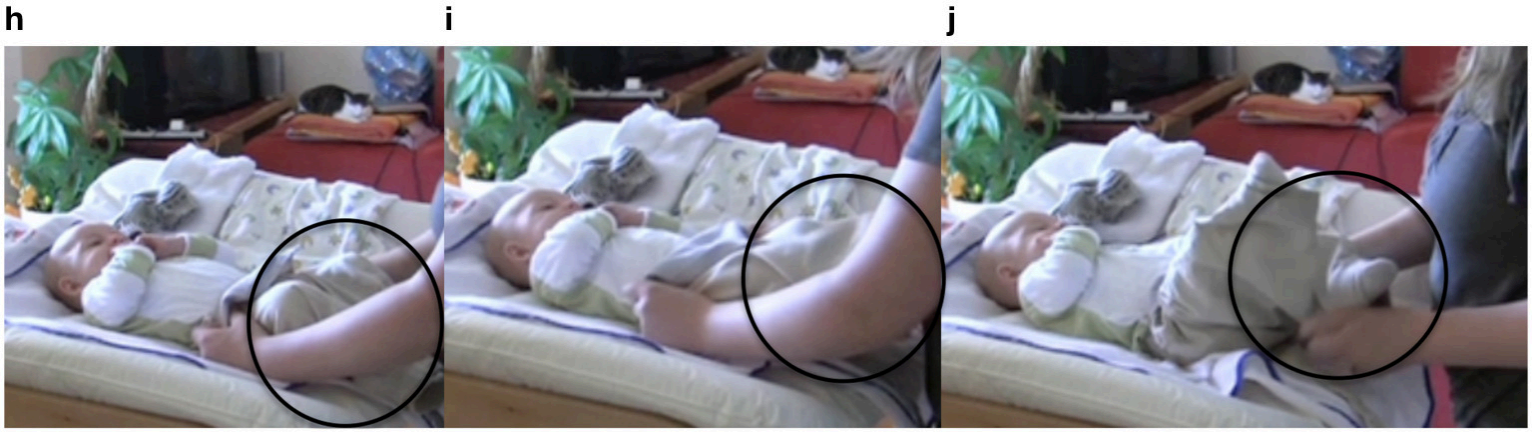
**Video grabs corresponding to Example 2, transcript lines 13–17**.

The mother then takes both legs and pushes them into the trousers. Having initiated this step of the dressing process, the mother pauses in line 13 and changes her bodily orientation and gaze from looking at the infant's legs to positioning herself in an upright position in the center of the changing table and engaging in eye contact with the infant. In this position, she pauses and waits (Figure 8h). In line 14, the infant vocalizes and subsequently stretches one leg through the trousers (Figure 8i). The mother responds by thanking the infant. In line 17, she grabs the other foot and pulls it through the leg of the trousers (Figure 8j) and thanks the infant again.


13 (2.8 s)^(h)^
14 I: <vocalization>
15 M: ^(i)^danke?
      *thank you?*
16 (1.8 s)
17 M: u::nd? (1 s) dan^(j)^ke?
      *a::nd? (1 s) thank you?*


This part of the sequence again illustrates the way in which the mother invites the infant to participate in the diapering activity. She pauses and creates slots for the infant's contribution (see also Rączaszek-Leonardi et al., 2013), at the same time reenacting culturally specific patterns—namely, saying “thank you” to another person for giving you something. The interesting detail in this sequence is that the mother says “thank you” in spite of the fact that she executes the action herself. Here, she is repeating the interactive pattern as if the infant had offered something, instilling again the value of collaboration and agentive participation.

Through multiple repetitions of situations like the ones above, utterances can be selectively linked to a specific constellation of aspects of the behavior of a speaker and a listener and of the situation they are immersed in. Thus, learning consists in the mother “moving” the baby into a cultural episode that she is enacting, and then, as the child begins to actively participate, in reinforcing proper joint actions. The value-realizing aspect of this process that we emphasize in this paper manifested themselves in those efforts made toward structuring the episode that seem persistent yet unnecessary and superfluous from the point of view of the sheer efficiency of a collective system in a given task (e.g., changing a diaper, dressing).

These illustrations pertain to the “enigmatic” in the quote recalled in Hodges and Baron: “*most episodes cannot be directly classified: they are enigmatic, having neither an explicit set of rules nor produced by well-established causal mechanisms*” (Harre and Secord, 1972, p. 12). Early in development, the non-specific mechanisms for creating a system (such as focusing on face and gaze following) are in place, yet the more specific values pertaining to “being together” in a situation (e.g., respecting each other) and maintaining social order in a particular culture can be imparted though such co-actions. The infant learns to co-act within social order in the “ecological way” by tuning to the actions of others as affordances for value-preserving actions and not by learning explicit social rules and norms. This is a possible way to normativity, as congruently with recent reformulations, social norms are understood as interaction patterns grounded in situated interpersonal relations (Brinck, 2014).

A question might arise as to the difference between values and social norms in the framework sketched above. Are caregivers enforcing social norms, or are the dyadic systems realizing socially important values? As one of the reviewers noted, what is expected by a group, culture or person is not necessarily equivalent to realizing values. Discussion of the difference between values and norms far exceeds the scope of this paper. One cannot take the easy way out and distinguish them on the basis of explicitness or being rule-based (e.g., constitutive rule-based norms) or more evasive (values). Not all social norms are formalized and conscious; some are just embodied in the ways “things are done” in a particular culture/society. From this perspective, social norms appear as instantiating values in a socially adequate way—congruent with other instantiations—choosing from among many possible constraining properties of a value those that are congruent within the normative system of a given culture/group.

## Conclusions

To further develop the framework in which linguistic utterances are treated as constraints on multisystem and multi-timescale dynamics—and to demonstrate its compatibility and affinity with the ecological approach to cognition—we sought to show the place of values and value-realization in the process of the emergence and use of linguistic constraints. Although the language-as-replicable-constraints framework has been useful so far mostly in task-oriented linguistic interactions, in which it seems to help in making predictions about the efficiency of coordinative systems, we hope to have demonstrated that this task-functionality is not the sole criterion for structure selection and stabilization. This paper aimed to show that the framework, far from being functionally mechanistic, inevitably encompasses multiple value realization by presenting interactions as being based in affordances.

The systemic approach to language as social coordination, along with the emphasis on the physical aspect of enacted values and the notion of timescales, helps to elucidate further the role of values and to guide the search for them. Some important values can be seen to stem from the requirements of holding a structured system together—and thus perhaps more can be explained by “biological accounts of natural selection” than we usually think. The mechanisms for preserving and realizing values through embodiment have been illustrated with examples from a developmental timescale, in which the behaviors of the caregiver, which were not directly necessary for the current activity of a dyad, can be seen as instilling values through the reenactment of cultural scripts.

The guidelines for the study of values within this “morality-in-motion” view, point to reading sets of values carefully off of multiple interactions, which reveal them in a subtle way. In our examples, these values included acknowledging the participation of the other in interaction, treating her/him as a responsible and independent agent, respecting the other's input, and mutually acknowledging the structures of interactive episodes, such as turn-taking. The best sources of data for such analyses seem to be the natural and quasi-natural situations of collaboration. Our examples pertained to the developmental timescale, but the on-line interactive episodes of adults are amenable to similar types of analysis, and several successful attempts at such analysis have appeared recently (Pedersen, 2012; Steffensen and Pedersen, 2014).

The above approach enables a deeper insight into the meanings that are co-created during participation in interactions by highlighting their embeddedness in larger fields of values. At the same time, it offers hints as to the values being realized. For this, however, the coordinative role of language has to come to the fore, which, in turn, seems better explained if language is not treated as a tool of “information exchange,” but rather as a perception-action system, enabling the formation of coherent, functional and flexible organizations with their own level of agency.

### Conflict of interest statement

The authors declare that the research was conducted in the absence of any commercial or financial relationships that could be construed as a potential conflict of interest.
